# Identification of quantitative trait loci associated with leaf rust resistance in rye by precision mapping

**DOI:** 10.1186/s12870-024-04960-6

**Published:** 2024-04-17

**Authors:** Mateusz Matuszkiewicz, Agnieszka Grądzielewska, Magdalena Święcicka, Alperen Ozturk, Monika Mokrzycka, Dolapo Igbari Aramide, Jie Song, Andrzej Kilian, Monika Rakoczy-Trojanowska

**Affiliations:** 1 Department of Plant Genetics, Breeding and Biotechnology, Institute of Biology, Warsaw, University of Life Sciences, Warsaw, Poland; 2 Educo BSH Ltd, Lublin, Poland; 3 Graduate School of Science and Technology, Niigata University, Niigata, Japan; 4 Department of Biometry and Bioinformatics, Institute of Plant Genetics Polish Academy of Sciences, Poznań, Poland; 5 Department of Botany, Faculty of Science, University of Lagos, Akoka, Lagos, Yaba Nigeria; 6 Diversity Arrays Technology, University of Canberra, Monana Street, Bruce, ACT 2617 Australia

**Keywords:** *Secale cereale*, Genome-Wide Association Study (GWAS), Immune response, Plant fungal pathogens, DArTseq markers, SilicoDArT markers

## Abstract

**Background:**

Leaf rust (LR) is among the most destructive fungal diseases of rye (*Secale cereale* L.). Despite intensive research using various analytical and methodological approaches, such as quantitative trait locus (QTL) mapping, candidate gene expression analysis, and transcriptome sequencing, the genetic basis of the rye immune response to LR remains unclear.

**Results:**

A genome-wide association study was employed to detect QTLs controlling the immune response to LR of rye. A mapping population, G38A, was constructed by crossing two inbred lines: 723 (susceptible to LR) and JKI-NIL-Pr3 (a donor of the LR resistance gene *Pr3*). For genotyping, SNP-DArT and silico-DArT markers were used. Resistance phenotyping was conducted by visual assessment of the infection severity in detached leaf segments inoculated with two isolates of *Puccinia recondita* f. sp. *secalis*, namely, 60/17/2.1 (isolate S) in the main experiment and 86/n/2.1_5x (isolate N) in the validation experiment, at 10 and 17 days post-infection (dpi), respectively.

In total, 42,773 SNP-DArT and 105,866 silico-DArT markers were included in the main analysis including isolate S, of which 129 and 140 SNP-DArTs and 767 and 776 silico-DArTs were significantly associated (*p* ≤ 0.001; − log_10_(*p*) ≥ 3.0) with the immune response to LR at 10 and 17 dpi, respectively. Most significant markers were mapped to chromosome 1R. The number of common markers from both systems and at both time points occupying common chromosomal positions was 37, of which 21 were positioned in genes, comprising 18 markers located in exons and three in introns. This gene pool included genes encoding proteins with a known function in response to LR (e.g., a NBS-LRR disease resistance protein-like protein and carboxyl-terminal peptidase).

**Conclusion:**

This study has expanded and supplemented existing knowledge of the genetic basis of rye resistance to LR by (1) detecting two QTLs associated with the LR immune response of rye, of which one located on the long arm of chromosome 1R is newly detected, (2) assigning hundreds of markers significantly associated with the immune response to LR to genes in the ‘Lo7’ genome, and (3) predicting the potential translational effects of polymorphisms of SNP-DArT markers located within protein-coding genes.

**Supplementary Information:**

The online version contains supplementary material available at 10.1186/s12870-024-04960-6.

## Background

Although rye (*Secale cereale* L.) is considered to be among the cereal crops most tolerant to environmental stresses, more than 30 diseases, including leaf rust (LR), infect this species. LR, which is caused by *Puccinia recondita* Roberge ex Desm. f. sp. *secalis* Miedaner, Klocke, Flath, Geiger & Weber (*Prs*), is among the most damaging diseases of rye, causing up to 40% yield losses [1].

Different molecular methods have been applied to examine the genetic background of the rye immune response to LR. Using a linkage mapping approach, 16 dominant *Pr* genes have been identified, namely *Pr1–5*, *Pr-d–f*, *Pr-i–l*, *Pr-n*, *Pr-p*, *Pr-r*, and *Pr-t* [2–4]. The genes *Pr1–Pr5*, *Pr-d–f*, *Pr-n*, *Pr-p*, and *Pr-r* are associated with resistance at both the seedling and the adult stages, indicating that they are all-stage resistance genes [3, 5]. Of the mentioned genes, *Pr1–5*, *Pr-d–f*, *Pr-n*, and *Pr-r* confer resistance to a broad range of single-pustule isolates [3, 6].

The majority of rye *Pr* genes have been mapped to individual chromosomes: *Pr3*, *Pr4*, *Pr5*, *Pr-i*, *Pr-k*, and *Pr-n* to chromosome 1R, *Pr-d* and *Pr-f* to 2RS, *Pr-j* and *Pr-l* to 4R, *Pr1* and *Pr-e* to 6R, and *Pr2* to 7RL [2, 3, 6–9]. The chromosomal localization of the genes *Pr-p*, *Pr-r*, and *Pr-t*, found in populations from Argentina, USA, and Russia, could not be resolved [3]. The results presented by Milczarski et al. [10] are not entirely consistent with those described above. Based on a mapping population derived from the recombinant inbred lines 541 (one parental component of the mapping population used in the present study) and Ot1-3, with the use of diversity array technology (DArT) markers, the authors identified ten LR resistance-related QTLs, of which four were mapped on chromosome 1R (two distributed on the short arm [1RS] and two on the long arm [1RL]), and the remaining six QTLs were mapped on chromosomes 3R (three QTLs; one on 3RS, one in the centromeric region, and the third QTL on 3RL), 4R (one QTL, in the centromeric region), and 5R (two QTLs, both in the centromeric region). The QTLs mapped to chromosome 5R have the strongest impact on resistance to LR. The authors emphasize, however, that each of the LR-QTLs was detected only in one year and in one location, which indicates that their effectiveness is highly dependent on the environment.

The results of the aforementioned studies were generated on the basis of interval mapping. Using a different approach, namely, genome-wide association study (GWAS; a strategy increasingly used for detection of resistance genes [11]), Vendelbo et al. [12, 13] mapped five LR resistance-associated QTLs on chromosome arms 1RS, 1RL, 2RL, 5RL, and 7RS using Gülzow-based elite hybrid rye breeding germplasm. Two QTLs located on chromosome arms 1RS and 7RS were of particular importance. The most important resistance-associated marker on chromosome arm 1RS was physically co-localized with molecular markers delimiting the previously characterized *Pr3* gene. The region on chromosome arm 7RS contained a large number of nucleotide-binding leucine-rich repeat (NLR) genes, one of which, provisionally denoted *Pr6*, was similar (at the protein level) to the wheat LR resistance gene *Lr1* situated on wheat chromosome arm 5DL. Rakoczy-Trojanowska et al. [14] used GWAS to identify the single DArT sequencing (DArTseq) marker (3363612|F|0–17:G > C-17:G > C) on chromosome 2R that was stably associated with LR resistance under field conditions.

In addition to the genes described above, we recently identified hundreds of other LR-related, differentially expressed genes using RNA-sequencing analysis of three rye inbred lines infected with compatible and incompatible strains of *Prs.* Among these genes were four wheat *Lr* gene orthologs (identified previously in the ‘Lo7’ genome [15]), namely, *ScLr1_3*, *ScLr1_4*, *ScLr1_8*, and *ScRga2_6*; the former two genes were located on chromosome 7R, whereas the latter two genes were of unknown location [16]. For now, however, it is not known whether any *ScLr1* variants are counterparts to the genes *Pr2* or *Pr6*, previously also assigned to chromosome 7R.

In the present study, which aimed to locate QTLs associated with LR resistance in rye, we used the DArTseq marker system for genotyping of the mapping population and a linear mixed model was developed based on observations included in the work of Bedo et al. [17] describing statistical machine learning (SML) for validation of QTLs. In principle, SML is unlike the interval mapping approach most commonly used for this purpose. The SML approach, first published in 2008 by Bedo et al. [17], relies on estimating the generalization performance of a QTL model. This is achieved by dividing the data into independent training and testing subsets. The training set is employed for model development, while the testing set is used for model evaluation. The authors, using a mapping population consisting of 94 F_1_-derived doubled-haploid plants from a cross between the barley cultivars ‘Steptoe’ and ‘Morex’, showed that this algorithm produces superior estimates of QTLs (for *α*-amylase, diastatic power, grain protein content, malt extract, heading date, height, lodging, and yield) than interval mapping and identifies QTLs with greater precision. Moreover, SML allows for identification of markers linked to QTLs without the need to construct a genetic map and, just as importantly, for reduction of the false-discovery rate. The machine learning approach has been repeatedly used and positively verified to identify QTLs [18] and expression QTLs [19], and as the basis for the AutoQTL analytical procedure [20] in plants.

The DArTseq marker system used for genotyping in the present work evolved from the microarray-based DArT platform developed more than 20 years ago [21] by combining restriction enzyme digestion with next-generation sequencing [22]. The DArTseq procedure generates two types of data: scores for presence/absence, called silico-DArTs (dominant markers, analogous to microarray DArTs, but extracted in silico from sequences obtained from genomic representations), and single-nucleotide polymorphisms (SNPs) in fragments present in the genomic representations (SNP-DArTs, co-dominant markers; https://www.diversityarrays.com/services/dartseq/dartseq-data-types/). To date, silico-DArTs and SNP-DArTs have been used in multiple studies of rye for development of SNP markers associated with selected agronomically important traits [14], construction of genetic maps and QTL identification [10, 23–25], targeting gene space [26], and determination and verification of phylogenetic relationships in the genus *Secale* [23, 27].

The aim of the present work was to better understand the genetic basis of rye resistance to LR by using a novel approach combining the principles of GWAS and linkage mapping.

## Methods

### Biological materials

The plant material consisted of (1) 329 plants of the F_2_/F_3_ mapping population G38A, the parents of which were the S_7_ inbred line 723 (bred at the West Pomeranian University of Technology, Szczecin, Poland), which is susceptible to LR, and the inbred line JKI-NIL-Pr3 (bred at the Julius Kühn-Institute Federal Research Centre for Cultivated Plants, Germany; kindly provided by Dr. Peter Wehling and Dr. Steffen Roux), a donor of the LR resistance gene *Pr3*; (2) the two parental lines; and (3) the LR-susceptible Polish rye cultivar ‘Konto’.

To obtain single-spore isolates of *Prs* that did not break the *Pr3* gene-based resistance (infected plants lacked this gene), 53 populations of LR were first collected from eight provinces in Poland in 2015–2017. Among them, nine populations (60/17/2.1, 67/17/2.2.1, 76/17/2_3x, 58/17/5.1, 60/17/2_3x, 34i/17/2/5.1, 76/17/2.1, 77/17/2.3, and 86/n/2.1_5x) were proved to be incompatible with donors of *Pr3* gene-based resistance to LR. From these nine populations, single-spore isolates were derived. Two of these nine isolates, namely, 60/17/2.1 and 86/n/2.1_5x (hereinafter referred to as isolates S and N, respectively) were characterized by segregation most similar to the Mendelian 1:2:1 ratio. The S isolate was used in the main experiment (involving the entire mapping population), whose data were used in QTL mapping, and the N isolate was used to verify the immune response of selected individuals (the verification experiment).

### Detached leaf test

The F_3_ seeds of the F_2_ G38A mapping population were sown on pallets in such a manner that one well contained the progeny of a single F_2_ plant. Ten-day-old seedlings were cut into approximately 1.5 cm fragments, which were then plated on four-well plates (Greiner Bio-One GmbH, Germany) filled with medium of the following composition: 3 g agar, 35 mg benzimidazole, and redistilled water up to 1 dm^3^. Each F_2_ plant was represented by 25–30 F_3_ seedlings. Leaf fragments of the LR-sensitive cultivar ‘Konto’ were placed around the lined leaf fragments of the G38A mapping population as a positive control for infection process (Fig. S1, S2).

Immediately after leaf fragments of the G38A mapping population and ‘Konto’ were placed on the plates, they were inoculated with isolates S and N. The isolate N was used only in the verification experiment. Spores were suspended in Novec™ 7100 engineered fluid (1 mg*cm^−3^). Each plate was sprayed four times. After inoculation, the plates were placed in a growth chamber under controlled conditions (18 °C, 16 h/8 h [light/dark] photoperiod, 50% relative humidity, and illumination intensity of 60 µmol m^−2^ s^−1^).

### Phenotyping of rye–LR interaction

Evaluation of the specificity of the plant–pathogen interaction was performed twice, at 10 days post-inoculation (dpi) and 17 dpi, using the 0–5 Murphy scale [28], where 0 = immune (no visible reaction), 1 = very resistant (chlorotic and necrotic flecking), 2 = resistant (minute uredinia, surrounded by chlorosis or necrosis), 3 = resistant to moderately resistant (small to medium-size uredinia, surrounded by chlorosis or necrosis), 4 = moderately resistant to moderately susceptible (medium to large uredinia, surrounded by chlorosis), and 5 = susceptible (large uredinia without chlorosis). The results of the disease-symptom evaluation are presented as the percentage of leaf fragments showing a given reaction. The phenotypes were described by “values used in the association analysis”, VAA (Table S1).

The disease-symptoms associated with a given *Prs* isolate and a given time point were treated as separate traits and analyzed separately. Thus, the following four traits were included in the mapping analysis: reaction to isolate S, 10 dpi (S_10) and to isolate S, 17 dpi (S_17) in the main experiment; and reaction to isolate N, 10 dpi (N_10) and to isolate N, 17 dpi (N_17) in the verification experiment.

### Genotype determination

Determination of the genotype of the plants included in the mapping population, i.e., whether a given plant was a dominant homozygote, recessive homozygote, or heterozygote, was based on the percentage of leaf fragments with a certain type of immune response in the 0–5 Murphy scale. Plants with a predominant proportion of leaf fragments in classes 0, 1, and/or 2 were classified as dominant homozygotes; plants with a predominant proportion of leaf fragments in classes 4 and/or 5 were categorized as recessive homozygotes; and plants in which leaf fragments were distributed in all or almost all immune response classes were denoted as heterozygotes.

### Molecular marker system

Genomic DNA for SNP-DArT (DArTseq) and silico-DArT genotyping was isolated from 329 plants of the G38A mapping population and the two parental lines using the Mag-Bind Plant DNA 96 Kit (Omega Bio-Tek, USA) in accordance with the manufacturer’s instructions. One hundred milligrams of ground leaf tissue was used for each analyzed plant. The isolated DNA was dissolved in 100 μl elution buffer. The DNA yield and purity were estimated using a spectrophotometer (NanoDrop 2000, Thermo Fisher Scientific, USA) and electrophoresed in 1% agarose gel stained with SimplySafe (Eurx, Poland). The DNA concentration was adjusted to 100 ng and then samples were sent to the Diversity Arrays Technology Pty Ltd. (Bruce, Australia), where genotyping was performed employing the DArTseq 1.0 technology developed by Cruz et al. [29] (Table S2 and S3).

Marker annotation was performed by mapping the sequences and chromosome positions to the Lo7 rye genome [15]. All gene names mentioned in the text are taken from Rabanus-Wallace et al. [15].

### GWAS

The values used in the association analysis were calculated using the following formula:

6 × value in column B/100 + 5 × value in column C/100 + 4 × value in column D/100 + 3 × value in column E/100 + 2 × value in column F/100 + 1 × value in column G/100. The values calculated are included in Table S1.

The linear mixed model (LMM) was formulated as follows:

$${{\varvec{y}}}_{{\varvec{i}}}={\varvec{\mu}}+{{\varvec{g}}}_{{\varvec{i}}}+{{\varvec{a}}}_{{\varvec{i}}}+{{\varvec{\varepsilon}}}_{{\varvec{i}}}$$

where *y*_*i*_ is the phenotype value of the *i*th line, *µ* is the overall mean, *g*_*j*_ is the fixed effect of the *j*th marker, *a*_*i*_ is the random effect of the *i*th line, and *ε*_*i*_ is the random error.

In the matrix notation, this model is as follows:

$$\mathbf{y}=\mathbf{X}{\varvec{\upbeta}}+\mathbf{Z}\mathbf{a}+{\varvec{\upvarepsilon}}$$

where **y** is the vector of the phenotypes, **β** = (β_0_, β_g_)’ is the vector of fixed effects, where β_0_ is the mean and β_g_ is the marker effect, **X** is the incidence matrix of fixed effects, which relates records to the marker genotypes (*g*) and mean, **a** is the vector of random line effects, **Z** is the incidence matrix of random effects, which relates records to the line effects, and **ε** is the vector of random errors.

This is a classic linear mixed model. The variable **a** models the genetic background of each line as a random effect with variance var(**a**) =$\boldsymbol A\sigma_a^2$  where **A** is the relationship matrix that can be estimated from the markers.

The ‘GWAS’ function in the R package ‘rrBLUP’ (https://cran.r-project.org/web/packages/rrBLUP/rrBLUP.pdf) implements the mixed model and was used to perform a genome-wide association analysis. The ‘GWAS’ function is equivalent to EMMAX [30] and calculates the score − log_10_(*p*-value) for each marker for the trait. If a marker had − log_10_(*p*-value) = 3, it was considered to be likely significantly associated with the trait.

A Manhattan plot for each trait was generated after applying the mixed model. The Manhattan plot represents the significance of the association between a marker and the trait being measured. The *Y*-axis is − log_10_(*p*-value), which represents the strength of association; the higher the point on the scale, the more strongly significant the association with the trait.

The mixed model approach results in the generation of a large number of correlated *p*-values (tests). Given the many single-point association analyses in a GWAS, adjustment of the *p*-values to correct for multiple testing is always a challenge, and was the focus of a further analysis step.

The GWAS analysis was implemented on the DArT KDCompute platform.

### Prediction of translational effects

The translational effects of SNPs (DArTseq markers) were predicted with the Ensembl Variant Effect Predictor [31].

## Results

### Distribution of phenotypic classes in the mapping population

**Fig. 1:**
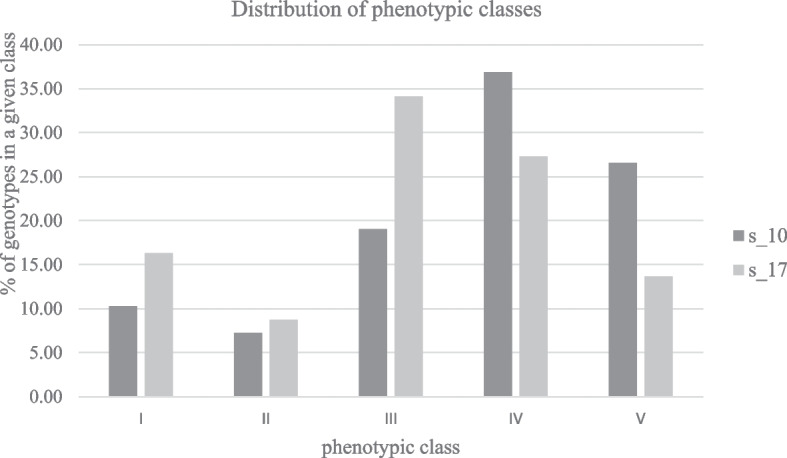
Distribution of phenotypic classes (described by VAA values) in G38A mapping population. Classes for S_10: I - 1.0 ÷ 2.0; II – 2.03 ÷ 2.97; III - 3.02 ÷ 4.0; IV – 4.01 ÷ 5.0; V – 5.06 ÷ 6.0. Classes for S_17: I - 1.0 ÷ 2.0; II – 2.14 ÷ 3.0; III - 3.05 ÷ 4.0; IV – 4.01 ÷ 5.0; V – 5.01 ÷  5.85 (Table S1)

In the main experiment involving the entire mapping population and the S isolate of *Prs*, the distribution of disease-symptom phenotypes deviated from the normal distribution, which resulted from under-representation of F_2_/F_3_ plants in the Values used in the Association Analysis (VAA) class II, both at 10 and 17 dpi. At the former time point, plants were most numerous in the class IV (VAA-4.01 ÷ 5), whereas at 17 dpi plants were most frequent in the class III (VAA—3.05 ÷ 4); (Fig. 1, Table S1).

The verification experiment performed using isolate N (which was of similar pathogenicity to that of the S isolate) confirmed the phenotypic assessments for all evaluated individuals of the mapping population. All results of the validation experiment are presented in Tables S1, S8, S9, S10, S11 and Figures S3, S4.

### Identification of SNP-DArT and silico-DArT markers associated with response to LR

A majority of markers included in the main analysis involving isolate S were assigned to Lo7 chromosomes (Table 1, S4, S5, S6, S7).

**Table 1:** Results of the genome-wide association analysis for the S isolate of *Puccinia recondita* f. sp. *secalis*

Marker type	Chromosome	No of markers	No (%) of markers associated with immune response to LR					
			Total		Significant at 0.001 > *p* ≤ 0.05*		Significant at *p* ≤ 0.001**	
			10 dpi	17 dpi	10 dpi	17 dpi	10 dpi	17 dpi
SNP-DArT	0R	2034	352 (17.31)	352 (17.31)	7 (0.34)	5 (025)	1 (0.05)	0 (0.00)
	1R	4179	892 (21.34)	891 (21.32)	216 (5.17)	221 (5.29)	107 (2.56)	115 (2.75)
	2R	4722	1047 (22.17)	1047 (22.17)	17 (0.36)	19(0.40)	2 (0.04)	2 (0.04)
	3R	4193	828 (19.75)	829 (19.77)	9 (0.21)	11 (0.26)	1 (0.02)	0 (0.00)
	4R	4927	959 (19.46)	959 (19.46)	5 (0.10)	11 (0.22)	1 (0.02)	0 (0.00)
	5R	4733	1046 (22.10)	1047 (22.12)	28 (0.59)	26 (0.55)	1 (0.02)	0 (0.00)
	6R	4691	914 (19.48)	911 (19.92)	12 (0.26)	12 (0.26)	0 (0.00)	0 (0.00)
	7R	4437	1012 (22.81)	1013 (22.83)	14 (0.32)	14 (0.32)	0 (0.00)	0 (0.00)
	NA	8857	1572 (17.75)	1559 (17.60)	57 (0.64)	54 (0.61)	16 (0.18)	23 (0.26)
	**Σ**	**42,773**	**8622 (20.16)**	**8608 (20.12)**	**365 (0.85)**	**373 (0.87)**	**129 (0.30)**	**140 (0.33)**
silico-DArT	0R	4040	1923 (47.60)	1920 (47.52)	33 (0.82)	40 (0.99)	12 (0.30)	8 (0.20)
	1R	7015	3554 (50.66)	3558 (50.72)	1010 (14.40)	911 (12.99)	552 (7.87)	519 (7.40)
	2R	8422	3987 (47.34)	3984 (47.30)	41 (0.49)	58 (0.69)	9 (0.11)	9 (0.11)
	3R	7285	3097 (42.51)	3095 (42.48)	53 (0.73)	65 (0.89)	4 (0.05)	7 (0.10)
	4R	9035	4295 (47.54)	4291 (47.49)	74 (0.82)	68 (0.75)	8 (0.09)	8 (0.09)
	5R	8536	4024 (47.14)	4018 (47.07)	116 (1.36)	77 (0.90)	5 (0.06)	7 (0.08)
	6R	8469	3865 (45.64)	3864 (45.63)	33 (0.39)	40 (0.47)	6 (0.07)	5 (0.06)
	7R	7673	3795 (49.46)	3794 (49.45)	45 (0.59)	72 (0.94)	8 (0.10)	6 (0.08)
	NA	45,391	14,847 (32.71)	14,873 (32.77)	536 (1.18)	545 (1.20)	163 (0.36)	207 (0.46)
	**Σ**	**105,866**	**43,387 (40.98)**	**43,397 (40.99)**	**1941 (1.83)**	**1876 (1.77)**	**767 (0.72)**	**776 (0.73)**

^*^ 3 <  − log_10_(*p*) ≥ 1.301029996; ** − log_10_(*p*) ≥ 3; 0R, chromosome Un (based on Rabanus-Wallace et al. [15]); *NA* markers not assigned

With regard to SNP-DArT markers, 365 and 373 markers were significantly (0.001 > *p* ≤ 0.05; 3 <  − log_10_(*p*) ≥ 1.301029996) associated with the response to LR at 10 and 17 dpi, respectively. In the case of silico-DArT markers, these values were 1941 and 1876, respectively. Applying a more stringent selection criterion, i.e., *p* ≤ 0.001, − log_10_(*p*) ≥ 3.0, the numbers of markers significantly associated with each trait were 129 (SNP-DArTs at 10 dpi), 140 (SNP-DArTs at 17 dpi), 767 (silico-DArTs at 10 dpi) and 776 (silico-DArTs at 17 dpi).

By far, the greatest number of markers significantly associated with all traits, both in the case of SNP-DArT and silico-DArT markers, were assigned to chromosome 1R; the proportion ranged from 48.56% for S_17 at 0.001 > *p* ≤ 0.05 to 82.95% for S_10 at *p* ≤ 0.001 (Table 2).

**Table 2:** Percentage of markers assigned to rye chromosome 1R

	0.001 > *p* ≤ 0.05		*p* ≤ 0.001	
	S_10	S_17	S_10	S_17
SNP-DArT	59.18	59.25	82.95	82.14
silico-DArT	52.04	48.56	71.97	66.88

For further detailed analysis, only markers assigned to chromosome 1R and significant at *p* ≤ 0.001 (hereinafter referred to as s_SNP-DArT and s_silico-DArT markers) were selected (Table S12). The results for markers with a *p*-value between 0.05 and 0.001 are shown in Table S13.

### Detailed characteristics of markers mapped to chromosome 1R—s_SNP-DArT markers

**Fig. 2:**
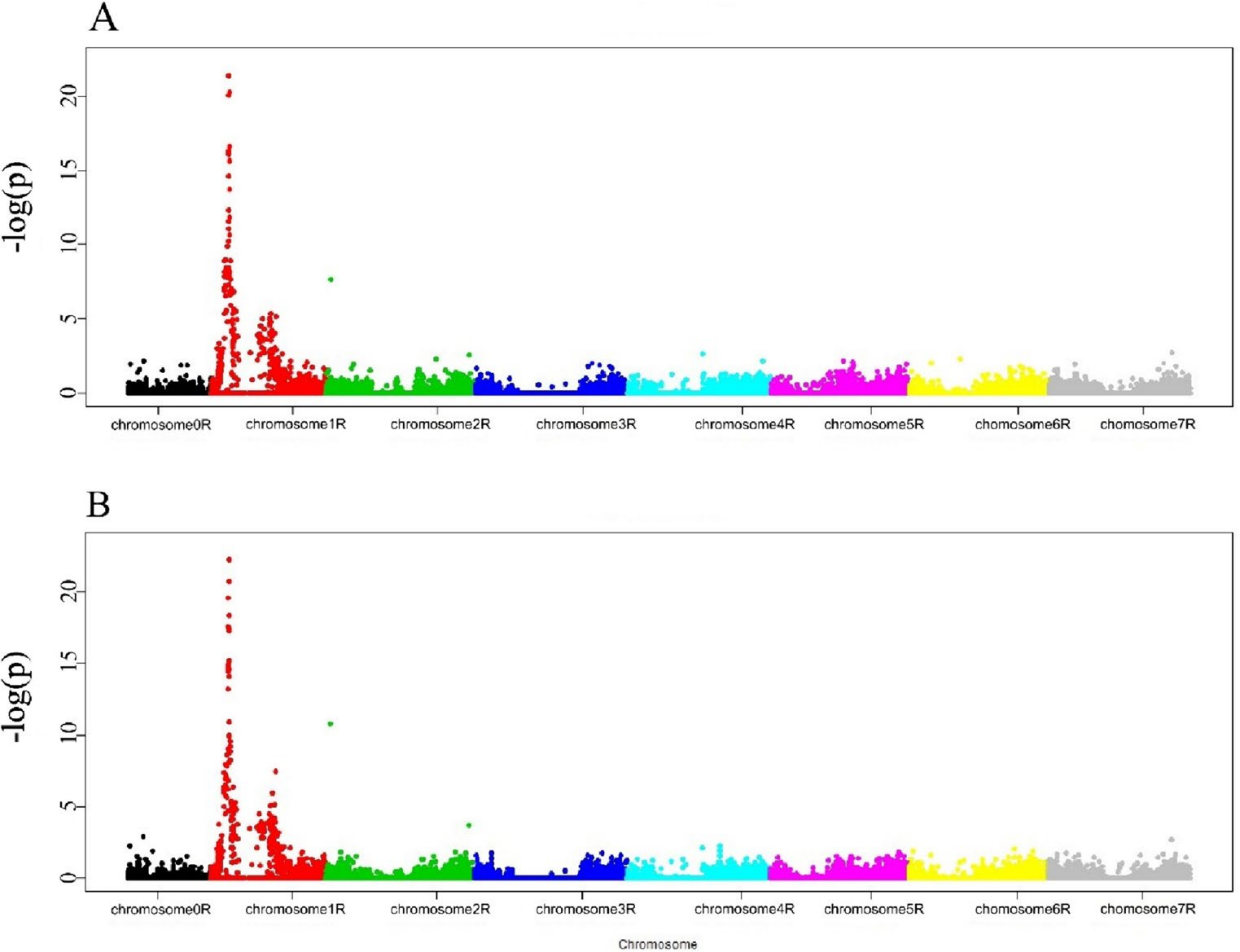
Manhattan plot visualizing SNP-DArT markers significantly associated with the immune response to *Prs* isolate S. a. 10 dpi. b. 17 dpi

In the Manhattan plot generated based on the SNP-DArT markers, at 10 dpi two clearly separated peaks/QTLs spanning from 53,241,837 to 164,974,087 (which corresponded to 1RS) and from 291,247,877 to 411,193,503 (which corresponded to 1RL), respectively, were observed (Fig. 2a, Tables S4, S12).

In general, the composition of QTLs at both time points was extremely similar. Of the 107 (2.56%) s_SNP-DArT markers associated with the S_10 trait, 64 were mapped to rye Lo7 genes [15]; most of these markers (53) were located in exons (Table 1, S4, S12). In the first peak/QTL, three s_SNP-DArT markers characterized by the highest − log_10_(*p*) values (≥ 20) were located in Lo7 genes encoding a NBS-LRR disease resistance protein-like protein (marker 5034809|F|0–32:G > C-32:G > C located in an exon), a desiccation-related protein PCC13-62 (marker 3365190|F|0–64:C > G-64:C > G located in an exon), and a phenazine biosynthesis PhzC/PhzF family protein (marker 3363069|F|0–46:G > C-46:G > C located in an exon). In the second peak/QTL, two s_SNP-DArT markers, namely, 3907484|F|0–52:C > T-52:C > T and 3360254|F|0–23:C > T-23:C > T, with the highest − log_10_(*p*) values (> 5) were located in rye Lo7 genes encoding a long-chain-alcohol oxidase (marker located in an exon) and folylpolyglutamate synthase (marker located in an intron), respectively. In addition to the 65 markers located in genes, six markers (four within the first QTL and two within the second QTL) were mapped to transposable elements (Table S12).

A total of 115 (2.75%) s_SNP-DArT markers were associated with the S_17 trait, including 68 mapped to the Lo7 genome; most of these markers (56) were located in exons (Table 1, S5, S12). The GWAS for S_17 showed the presence of two peaks/QTLs on chromosome 1R, covering the regions from 53,241,837 to 164,974,087 (identical as for S_10) and from 247,907,747 to 426,929,972 (partially overlapping with the second QTL for S_10, but with a range larger by more than 59 Mb) (Fig. 2b, Table S12).

In the first peak/QTL two markers characterized by the highest − log_10_(*p*) values (≥ 20) were located within exons of genes encoding a desiccation-related pcC13-62 protein (marker 3365190|F|0–64:C > G-64:C > G) and a pathogenesis-related thaumatin family protein (marker 3594357|F|0–29:A > C-29:A > C). A third marker with − log_10_(*p*) ≥ 20, associated with a NBS-LRR disease resistance protein-like protein at 10 dpi, in the case of S_17 was characterized by a slightly lower, but still extremely high, − log_10_(*p*) value of 19.59605099 (Table S12). In the second peak/QTL, two s_SNP-DArT markers, namely, 5788995|F|0–62:T > G-62:T > G and 3363086|F|0–40:T > C-40:T > C, were associated most strongly with genes encoding folylpolyglutamate synthase (the marker was located in an intron) and carboxypeptidase (the marker was located in an exon), respectively (Table S12).

As in the case of S_10, the same six markers were located within transposable elements (Table S12).

In addition to the aforementioned markers associated with the immune response at extremely high levels of significance, at both time points and in both QTLs, the presence of markers associated with the analyzed traits was detected at slightly lower (but still high) levels of significance in genes typical for the immune response, such as those encoding a receptor-like kinase, endoglucanase, cellulose synthase-like protein, and a MYB protein or myosin (Table S12).

**Fig. 3:**
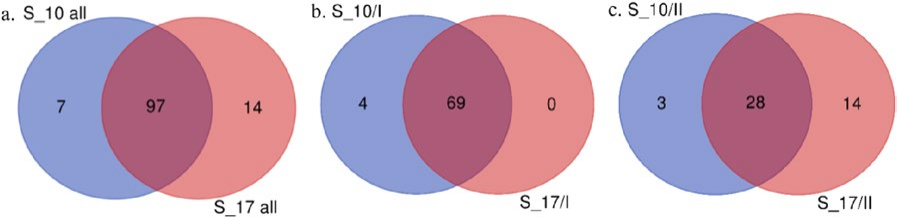
Common and unique significant SNP-DArT markers. Venn diagrams represents the number of common and unique s_SNP-DArT markers for 10 and 17 days post-inoculation (dpi). **a** Both QTLs on chromosome 1R; **b** the first QTL on chromosome 1R; **c** the second QTL on chromosome 1R. The diagrams were generated using an online tool (https://bioinformatics.psb.ugent.be/webtools/Venn/). S_10 all referred to s_SNP-DArTseq markers in both QTLs, 10 dpi; S_17 all referred to s_SNP-DArTseq markers in both QTLs, 17 dpi; S_10/I referred to s_SNP-DArTseq markers in the first QTL, 10 dpi; S_17/I referred to s_SNP-DArTseq markers in the first QTL, 17 dpi; S_10/II referred to s_SNP-DArTseq markers in the second QTL, 10 dpi; S_17/II referred to s_SNP-DArTseq markers in the second QTL, 17 dpi

Ninety-seven s_SNP-DArT markers were common to both time points, whereas 7 and 14 markers were unique for 10 dpi and for 17 dpi, respectively (Fig. 3a, Tables S12, S14). Within the first peak/QTL, 69 s_SNP-DArT markers were common to both time points of which four were unique for 10 dpi and no markers were unique for 17 dpi (Fig. 3b, Tables S12, S14). In the case of the second QTL, 28 markers were common to both time points of which three and 14 markers were unique for 10 dpi and 17 dpi, respectively (Fig. 3c, Tables S12, S14).

### Detailed characteristics of markers mapped to chromosome 1R—s_silico-DArT markers

**Fig. 4:**
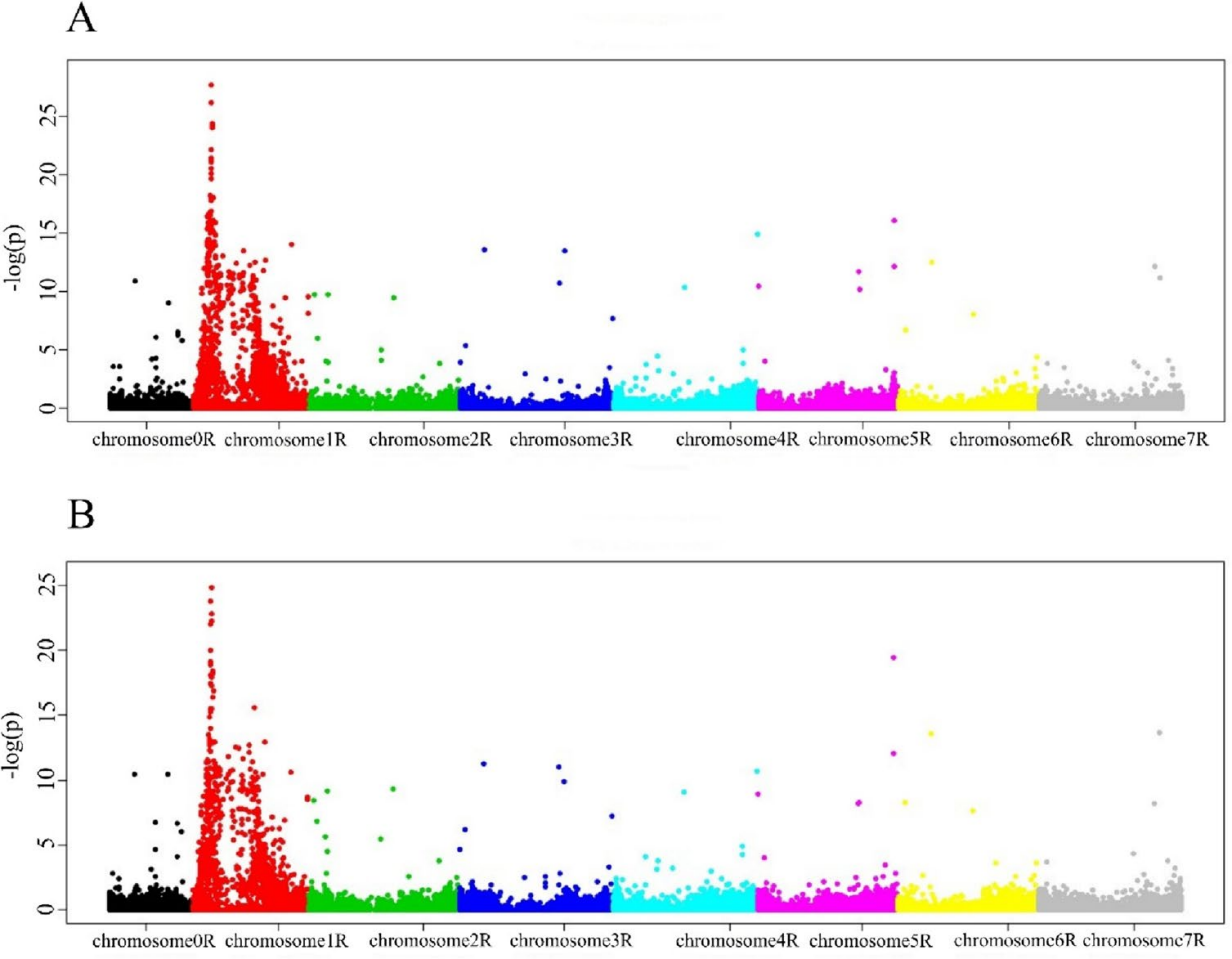
Manhattan plot visualizing silico-DArT markers significantly associated with the immune response to *Prs* isolate S. a. 10 dpi. b. 17 dpi

As for the s_SNP-DArT markers, two QTLs on chromosome 1R (in the ranges from 30,347,446 to 185,495,011 and 204,544,065 to 720,560,882) were associated with silico-DArT markers at 10 dpi, but the distance between the markers was less pronounced than for the s_SNP-DArT markers (Fig. 4a, Tables S6, S12).

In total, 552 (7.87%) of the s_silico-DArT markers were associated with the S_10 trait of which 220 markers were located in Lo7 genes and 172 of these in exons (Tables S6, S12). The gene-assigned s_silico-DArT markers with the highest − log_10_(*p*) values (> 20) for the first QTL were as follows: 3,342,055, 3,342,250, 3,342,695, 3,342,810, 3,342,996, 3,343,110, and 3,343,673 were located in exons of genes encoding a NBS-LRR disease resistance protein-like protein, pathogenesis-related thaumatin family protein, cytochrome P450, serine/threonine-protein kinase ATM, replication protein A 70 kDa DNA-binding subunit, invertase/pectin methylesterase inhibitor family protein, and CHUP1 protein, respectively. Most of these genes were associated with the immune response to fungal pathogens. Of the markers for the second QTL, several were located within genes associated with the response to pathogens; these included, e.g., genes encoding a myosin heavy chain-related protein (marker 5,044,425 located in an intron), MYB family transcription factor-like protein (marker 7,062,938 located in an exon; and marker 5,043,320 at 377,042,108 located in an intron), and carboxypeptidase (marker 5,039,638 located in an intron) (Table S12).

At the second time point, two QTLs (in the same ranges as those for 10 dpi) were generated by the GWAS analysis (Fig. 4b). In total, 519 (7.40%) silico_SNP-DArT markers were associated with the S_17 trait, comprising 207 markers located in genes of which 162 markers were located in exons (Tables S7, S12).

**Fig. 5:**
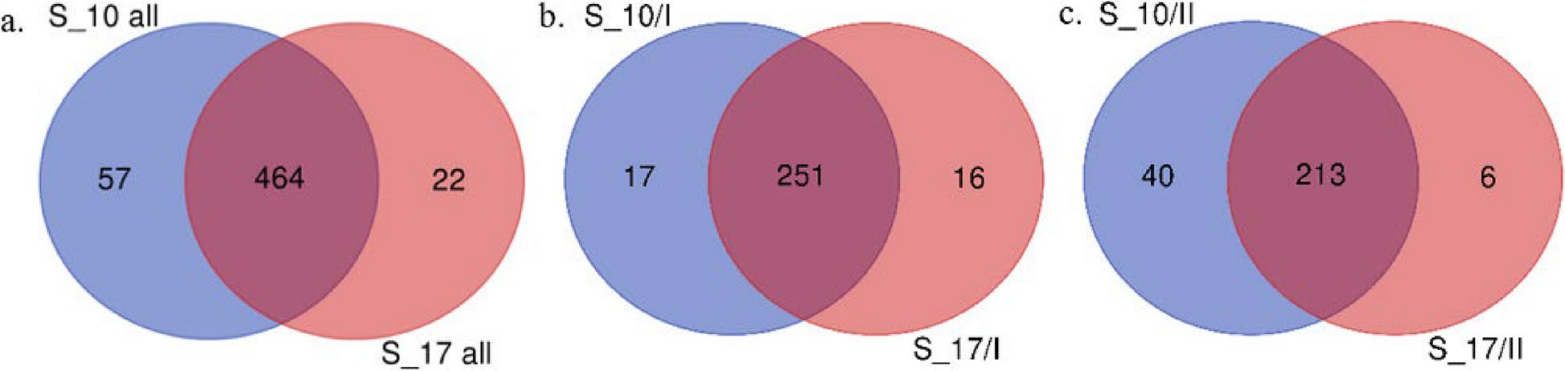
Common and unique significant silico-DArT markers. Venn diagrams representing numbers of common and unique s_silico-DArT markers for 10 and 17 dpi. a. Both QTLs on chromosome 1R; b. the first QTL on chromosome 1R; c. the second QTL on chromosome 1R. The diagrams were gener,ed with an online tool (https://bioinform.ics.psb.ugent.be/webtools/Venn/). S_10 all referred to s_silico-DArT markers in both QTLs, 10 dpi; S_17 all referred to s_silico-DArT markers in both QTLs, 17 dpi; S_10/I referred to s_silico-DArT markers in the first QTL, 10 dpi; S_17/I referred to s_silico-DArT markers in the first QTL, 17 dpi; S_10/II referred to s_silico-DArT markers in the second QTL, 10 dpi; S_17/II referred to s_silico-DArT markers in the second QTL, 17 dpi

In total, 464 s_silico-DArT markers were common for both time points, and 57 and 22 markers were unique for 10 dpi and for 17 dpi, respectively (Fig. 5a, Tables S12, S14). In the first QTL, 251 s_silico-DArT markers were common for both time points, and 17 and 16 markers were unique for 10 dpi and for 17 dpi, respectively (Fig. 5b, Tables S12, S14). For the second QTL, 213 markers were common for both time points, and 40 and 6 markers were unique for 10 dpi and for 17 dpi, respectively (Fig. 5c, Tables S12, S14).

### Common positions of significant SNP-DArT and silico-DArT markers

**Fig. 6:**
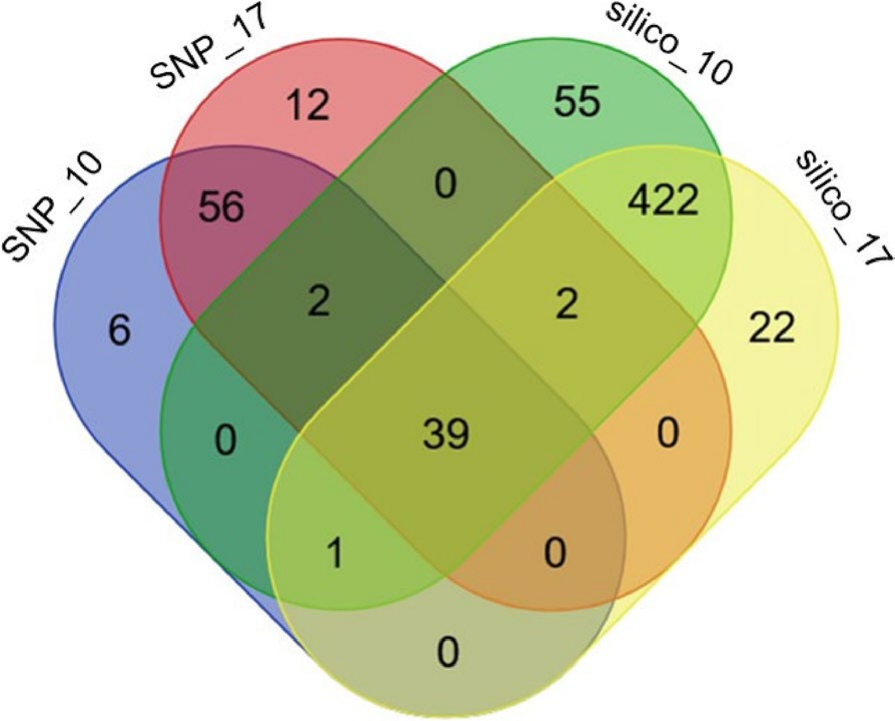
Common significant SNP-DArTs for 10 dpi and 17 dpi for both QTLs. Venn diagram represents the numbers of common and unique s_SNP-DArT and s_silico-DArT markers for 10 and 17 dpi. The diagram was generated using an online tool (https://bioinformatics.psb.ugent.be/webtools/Venn/). SNP_10 referred to s_SNP-DArTseq markers, 10 dpi; SNP_17 referred to s_SNP-DArTseq markers, 17 dpi; silico_10 referred to s_silico-DArT markers, 10 dpi; silico_17 referred to s_silico-DArT markers, 17 dpi

Thirty-nine markers from both marker systems and at both time points occupied common chromosomal positions on chromosome 1R. Of these markers, 21 were located in genes (hereinafter referred to as the “common 21 pool”), comprising 18 markers located in exons and three markers located in introns. In addition, two common markers were assigned to transposable elements. The remainder of the markers were located in intergenic regions (Fig. 6, Table 3, S12, S14).

**Table 3:** Markers occupying identical chromosomal positions at both time points: 10 and 17 dpi. Table presents “common 21 pool” of significant SNP-DArT and silico-DArT markers that shared common chromosomal positions for two time points.*) First, reference allele; second, SNP allele. **) Marker genotype in LR-resistant lines (VAA ≥ 5; Table S1) and LR-susceptible lines (VAA ≤ 2.65; Table S1)

Marker position/QTL	Gene ID	Encoded protein	Location	*p*-value				SNP-DArT marker*	Marker genotype res/sus**
				s_SNP-DArTs, S_10	s_SNP-DArTs, S_17	s_silico-DArTs, S_10	s_silico-DArTs, S_17		
82,476,223/I	SECCE1Rv1G0011790.1	GDSL esterase/lipase	exon (1/2)	6.912	6.347	12.525	10.247	7467766|F|0–6:T > G	GG/TT
82,803,484/I	SECCE1Rv1G0011840.1	protein phosphatase 2C-like protein	intron (1/3)	7.855	6.010	15.311	10.476	3730018|F|0–57:A > G	AA/GG
82,809,999/I	SECCE1Rv1G0011850.1	RNA polymerase II subunit B1 CTD phosphatase RPAP2	exon (1/6)	8.903	7.409	16.432	11.444	3362858|F|0–20:G > C	CC/GG
86,006,833/I	SECCE1Rv1G0012070.1	dehydrogenase	exon (2/3)	6.978	6.384	11.419	4.036	5203314|F|0–14:C > T	CC/TT
86,566,724/I	SECCE1Rv1G0012110.1	peptide chain release factor 1	exon (1/2)	9.003	7.953	15.849	11.601	3603211|F|0–20:C > G	CC/GG
93,659,576/I	SECCE1Rv1G0012540.1	lecithin-cholesterol acyltransferase-like 1	exon (1/1)	7.899	6.349	16.657	13.489	3597912|F|0–20:C > T	CC/TT
99,955,159/I	**SECCE1Rv1G0013000.1**	DNA repair helicase	exon (11/11)	4.803	5.678	18.194	14.852	3596033|F|0–14:T > C	TT/CC
100,735,094/I	**SECCE1Rv1G0013110.1**	endoglucanase	exon (7/7)	8.091	8.574	7.376	9.133	3596652|F|0–30:A > G	GG/AA
108,660,456/I	**SECCE1Rv1G0013840.1**	invertase/pectin methylesterase inhibitor family protein	exon (1/1)	16.272	14.424	21.278	18.861	3591362|F|0–11:A > C	AA/CC
109,130,631/I	**SECCE1Rv1G0013960.1**	protein CHUP1	exon (1/2)	16.069	14.752	20.085	5.391	3345552|F|0–36:A > G	GG/AA
111,147,729/I	**SECCE1Rv1G0014220.1**	NBS-LRR disease resistance protein-like protein	exon (1/1)	21.381	19.596	26.171	5.160	5034809|F|0–32:G > C	CC/GG
114,605,663/I	**SECCE1Rv1G0014450.1**	pathogenesis-related thaumatin family protein	exon (2/2)	16.633	20.705	24.255	7.960	3594357|F|0–29:A > C	CC/AA
119,156,676/I	**SECCE1Rv1G0014990.1**	phenylalanine ammonia-lyase	exon (1/1)	8.951	9.206	11.689	4.436	3587847|F|0–44:C > A	AA/CC
132,257,012/I	**SECCE1Rv1G0015900.1**	receptor-like kinase	exon (1/1)	4.925	4.905	12.837	10.778	3349900|F|0–20:T > C	TT/CC
132,257,078/I	**SECCE1Rv1G0015900.1**	receptor-like kinase	exon (1/1)	4.114	3.070	14.791	11.324	3344187|F|0–12:G > C	CC/GG
133,009,915/I	**SECCE1Rv1G0015990.1**	MYB-related transcription factor	intron (1/2)	3.480	3.508	10.719	6.893	3899895|F|0–56:G > A	AA/GG
135,177,129/I	**SECCE1Rv1G0016170.1**	ring finger protein	exon (1/1)	4.692	4.597	13.963	5.013	75511600|F|0–32:T > G	TT/GG
140,611,121/I	SECCE1Rv1G0016670.1	carboxyl-terminal peptidase	exon (5/5)	5.659	6.333	9.342	8.507	5503350|F|0–5:G > A	AA/GG
140,893,901/I	SECCE1Rv1G0016710.1	leucine-rich repeat receptor-like protein kinase family	exon (1/1)	6.821	4.463	12.039	10.634	3353307|F|0–11:T > C	CC/TT
380,571,002/II	SECCE1Rv1G0027270.1	folylpolyglutamate synthase	intron (2/15)	5.367	5.049	8.720	8.538	3360254|F|0–23:C > T	CC/TT
404,779,809/II	SECCE1Rv1G0028640.1	kinase family protein***	exon (13/13)	3.091	4.214	3.855	3.364	3596868|F|0–24:T > C	CC/TT

***) Serine/threonine kinase (https://plants.ensembl.org/Secale_cereale/Transcript/ProteinSummary?db=core;g=SECCE1Rv1G0028640;r=1R:404779782-404784403;t=SECCE1Rv1G0028640.1)

**Bold** text highlights genes in the region between 96.7 and 137.6 Mb flanked by markers co-segregating with the *Pr3* gene, indicated by Vendelbo et al. [13]

The highest − log_10_(*p*) values (> 20) were observed for the following markers: s_silico-DArT at 10 dpi within genes encoding an invertase/pectin methylesterase inhibitor family protein, CHUP1 protein, NBS-LRR disease resistance protein-like protein, and pathogenesis-related thaumatin family protein; s_SNP-DArT at 10 dpi within the gene encoding a NBS-LRR disease resistance protein-like protein; and s_SNP-DArT at 17 dpi within the gene encoding a pathogenesis-related thaumatin family protein. Except for two genes in which markers for the second QTL were localized (*SECCE1Rv1G0027270.1* and *SECCE1Rv1G0028640.1* coding for folylpolyglutamate synthase and a kinase family protein, respectively), in all remaining genes markers for the first QTL were localized. Among the 18 markers localized in gene exons, two markers (3349900|F|0–20:T > C-20:T > C and 3344187|F|0–12:G > C-12:G > C; *SECCE1Rv1G0015900.1*) were located within the same gene, namely, *SECCE1Rv1G0015900.1* coding for a receptor-like kinase. Two intron-localized markers (3730018|F|0–57:A > G-57:A > G and 3899895|F|0–56:G > A-56:G > A) were located in the first intron of genes coding for a protein phosphatase 2C-like protein and a MYB-related transcription factor, respectively, whereas the marker 3360254|F|0–23:C > T-23:C > T was located in the second exon of the gene encoding folylpolyglutamate synthase.

In the case of nine gene-localized markers, the homozygote for the reference allele determined the LR-resistant phenotype, whereas the remaining 11 markers were associated with the homozygote for the alternative allele (Table 3). Data for the complete set of genes are provided in Table S12.

### Translational effect of SNPs located in genes

The ten SNPs located in exons of 18 genes from the “common 21 pool” had a low translational impact (i.e., synonymous protein variant formation without an amino acid change), seven SNPs had a moderate impact (mis-sense protein variant formation that resulted from different codons and, therefore, translation of a different amino acid), and only one SNP had a high impact with loss of the stop codon. This specific polymorphism (A/C) was located in the exon of the gene *SECCE1Rv1G0014450.1* encoding a pathogenesis-related thaumatin family protein; the effect of stop-codon loss was determined by the mutant genotype, CC, present in the LR-resistant rye lines. All three intron variants had modifier effects, i.e., the splicing process was not affected (Tables 3 and 4).

**Table 4:** Translational effect of single-nucleotide polymorphisms (SNPs) located in the genes from “common 21 pool”

SNP-DArT marker^a^	Consequence	Impact	cDNA position	CDS position	Protein position	Amino acids	Codons	Strand
7467766|F|0–6:T > G	missense variant	mod	59	59	20	Y/S	tAc/tCc	-1
3730018|F|0–57:A > G	intron variant	mof	-	-	-	-	-	1
3362858|F|0–20:G > C	synonymous variant	low	522	522	174	G	ggC/ggG	-1
5203314|F|0–14:C > T	synonymous variant	low	390	390	130	A	gcC/gcT	1
3603211|F|0–20:C > G	missense variant	mod	384	384	128	E/D	gaG/gaC	-1
3597912|F|0–20:C > T	missense variant	mod	856	856	286	V/M	Gtg/Atg	-1
3596033|F|0–14:T > C	synonymous variant	low	2232	2232	744	T	acA/acG	-1
3596652|F|0–30:A > G	synonymous variant	low	1017	1017	339	S	tcT/tcC	-1
3591362|F|0–11:A > C	synonymous variant	low	420	420	140	A	gcT/gcG	-1
3345552|F|0–36:A > G	missense variant	mod	701	701	234	M/T	aTg/aCg	-1
5034809|F|0–32:G > C	missense variant	mod	177	177	59	E/D	gaG/gaC	1
3594357|F|0–29:A > C	stop lost	high	553	553	185	*/E	Tag/Gag	-1
3587847|F|0–44:C > A	missense variant	mod	681	681	227	E/D	gaG/gaT	-1
3349900|F|0–20:T > C	synonymous variant	low	1551	1551	517	N	aaT/aaC	1
3344187|F|0–12:G > C	missense variant	mod	1580	1580	527	S/T	aGc/aCc	1
3899895|F|0–56:G > A	intron variant	mof	-	-	-	-	-	1
75511600|F|0–32:T > G	synonymous variant	low	495	495	165	A	gcT/gcG	1
5503350|F|0–5:G > A	synonymous variant	low	228	228	76	H	caC/caT	-1
3353307|F|0–11:T > C	synonymous variant	low	2286	2286	762	L	ctT/ctC	1
3360254|F|0–23:C > T	intron variant	mof	-	-	-	-	-	-1
3596868|F|0–24:T > C	synonymous variant	low	1368	1368	456	V	gtA/gtG	-1

^a^First, reference allele; second, alternative allele. mod, referred to moderate impact; mof referred to modifier impact; L referred to low impact. Marker location and gene ID are given in Table 3

Among the remaining genes containing s_SNP-DArTseq markers, only the SNP T/C (in the marker 3363086|F|0–40:T > C-40:T > C), located in the *SECCE1Rv1G0016320.1* gene encoding a haloacid dehalogenase-like hydrolase superfamily protein, may have a strong translational effect. Similar to the *SECCE1Rv1G0014450.1* gene, the effect of stop-codon loss was determined by the recessive allele present in LR-resistant lines (Tables S13, S15).

The search for rye genes conferring resistance to LR has been ongoing for more than 20 years, but our current knowledge, and especially the number of identified and characterized resistance genes, remains limited. Much of the research in this field published to date has aimed to identify regions/genes on genetic maps [6–8]. Of the 16 genes identified in this manner, of profound importance is the *Pr3* locus on chromosome 1R, which carries many genes controlling agronomically important traits but mainly involved in tolerance to abiotic and biotic stresses, including LR [32, 33].

### Identification of LR associated QTLs

To verify previous results and, above all, expand on existing knowledge, we identified QTLs for the immune response to LR of rye, but using a novel approach. The present study employed a combination of two highly effective tools: the DArTseq platform and LMM procedure based on SML described by Bedo et al. [17]. While DArTseq markers have been used in previous studies on rye (as outlined in the Introduction), including identification of QTLs and SNP markers associated with LR resistance [10, 14], the effectiveness of SML-like method has not been verified previously in this species. Nevertheless, machine learning has been shown to be a powerful and extremely precise tool in many fields. Bedo et al. [17] compared multiple methods for detection of QTLs in barley (for the following traits: malting quality traits, heading date, plant height, lodging, pubescent leaves, grain protein content, and yield), namely, composite interval mapping, Bayesian interval mapping, and single marker regression. The authors reported that SML generates narrower peaks than the other methods, thereby allowing QTL identification with greater precision. We also performed a comparative analysis for 105 DArTseq markers from chromosome 1R using the CIM method, which allowed the identification of only one peak spanning a very wide range – 161,174 cM, corresponding to 717,370,020 bp and not two QTLs covering much narrower areas, than when using LMM (from 53,241,837 bp to 164,974,087 bp and from 291,247,877 bp to 411,193,503 bp, respectively); Table S16.

### Characteristics of QTLs from chromosome 1R

In the present study, we focused on the rye genomic region that includes the *Pr3* gene. We used a F_2_/F_3_ mapping population that was developed by crossing two inbred lines: JKI-NIL-Pr3, a donor of the *Pr3* gene; and 723, an inbred line that lacks the *Pr3* gene. Phenotypic segregation deviated from the expected normal distribution owing to under-representation of lines in the VAA class 2.01–3 but slight overrepresentation of lines in the class 1–2. This might be caused by two factors: (1) imprecise phenotyping in some cases caused by superinfections with other pathogens, and (2) the parents of the mapping population were not characterized by extreme values for the S_10 and S_17 traits.

Based on previous research findings [12, 13] and taking into account that the inbred line JKI-NIL-Pr3 carried the *Pr3* gene, we expected to identify one region on chromosome 1R with markers located around the NBS gene cluster. We detected two distinct QTLs in all four GWAS analyses performed; the first QTL was detected on 1RS, and the second QTL on 1RL, of chromosome 1R. More than half (in the case of s_SNP-DArTs) and almost half (in the case of s_silico-DArTs) of the markers were located in genes, and mostly in exons. Although the gene-located s_silico-DArT markers were present in both peaks, they were substantially more abundant (by more than five times) in the first peak. More details on the selected genes containing markers significantly associated with the S_10 and S_17 traits for both QTLs are presented later in the discussion.

### Could *SECCE1Rv1G0014220.1* gene be considered a good candidate for *Pr3* candidate?

Interestingly, in the Lo7 genomic block spanning from 101 to 117 Mb that showed the strongest association with LR resistance in the GWAS analysis and in which Vendelbo et al. [13] detected as many as five NBS-NLR genes, we located only one NBS-LRR-encoding gene (*SECCE1Rv1G0014220.1*) containing s_SNP-DArTseq and s_silico-DArT markers located in position 111,147,729 Mb (Table S12). This position was identical to or very close to the position of one of two NBS-NLR paralogs in the Lo7 genome that showed 83.5%–85.1% sequence similarity to RenRS5_3, a candidate LR resistance gene detected by Vendelbo et al. [13]. In the present study, however, SNP markers located in the gene *SECCE1Rv1G0014220.1* were associated with LR resistance with extremely high probability (− log_10_(*p*) > 19), thus much higher than in the study of Vendelbo et al. [13] in which none of the markers exhibited a *p*-value above the Bonferroni significance threshold of − log_10_(*p*) = 6.72 (nevertheless, it should be taken into account that they used a natural population rather than a biparental population, which may explain the difference between their results and ours). Therefore, in our opinion, *SECCE1Rv1G0014220.1* should be considered to be among the best *Pr3* gene candidates conferring resistance to LR. However, the role of this gene may be related to down-regulation rather than up-regulation of its expression, as suggest results of preliminary RT-qPCR analyzes employing four rye inbred lines – two susceptible (Lo7 and L318) and two resistant (SE8 and SE212) to LR, infected with 1.1/6 isolate of *Prs* (Fig. S5a,b; Table S17). Also in our previous work, similar relationship was observed for several variants of the *ScLr1* gene belonging to the NBS-LRR family, which were down-regulated after infection with *Prs* [16].

The results of the present analysis differ almost entirely from those reported by Milczarski et al. [10] who detected four QTLs on chromosome 1R, two on 1RS (at positions 39.4 Mbp and 54.8 Mbp) and two on 1RL (at positions 154.7 Mbp and 240.1 Mbp). The marker 5225086|F|0–7:T > G-7:T > G (associated with LR resistance at a relatively low but still statistically significant − log_10_(*p*) value of 1.96457369) was the only SNP-DArTseq marker that was located near the second QTL on chromosome 1RS detected by Milczarski et al. [10] (Table S13). Unfortunately, this marker has not been mapped to a Lo7 gene. The differences between the present results and those of Milczarski et al. mainly reflect the disparate experimental conditions: first, Milczarski et al. assessed LR resistance under field conditions, and second, the causative agent of LR used by Milczarski et al. was a mixture of spores of many undefined *Prs* strains. It is also worth noting that the QTLs for LR resistance identified by Milczarski et al. [10] in 2013 did not overlap with those detected in the following year (2014).

As noted above, apart from the gene *SECCE1Rv1G0014220.1*, none of the other four NBS-LRR genes identified by Vendelbo et al. [13] were detected in the present analysis, neither in the block spanning from 101 to 117 Mb showing the strongest association with LR resistance in the GWAS analysis, nor in in the region between 96.7 and 137.6 Mb flanked by markers co-segregating with the *Pr3* gene. Instead, we identified in this area many other markers associated with LR resistance with extremely high probabilities, located in genes important from the perspective of the immune response to diseases caused by rust fungi; some of these genes contained s_SNP-DArT and s_silico-DArT markers located in positions common to all four GWAS analyses (highlighted in bold in Table 3).

### Markers located in Lo7 genes

The pool of both types of markers located in common positions significantly associated with the S_10 and S_17 traits amounted to 39 markers, 21 of which were located in genes, including 18 markers within exons. Notably, two markers forming a second QTL, *SECCE1Rv1G0027270.1* and *SECCE1Rv1G0028640.1*, were located in genes coding for folylpolyglutamate synthase and a kinase family protein, respectively; the former marker was located in an intron and the latter marker in an exon. Many of these 21 genes, such as those coding for a NBS-LRR disease resistance protein-like protein, leucine-rich repeat receptor-like protein kinase family protein, pathogenesis-related thaumatin family protein, phenylalanine ammonia-lyase, endoglucanase, MYB-related transcription factor, kinase (serine/threonine) family protein, and GDSL esterase/lipase ([34–41], respectively), have been proven to play a role in the immune response to rust fungi. One such gene, *SECCE1Rv1G0013110.1*, encoding an endoglucanase (to which marker 3596652|F|0–30:A > G-30:A > G has been mapped), has previously been observed to be differentially expressed (highly up-regulated; log_2_ estimated fold change = 4.7) in the rye inbred line L318 infected with compatible *Prs* strains [41]. Given the high scores for the marker (*p*-value) and the gene (log_2_ estimated fold change), we suggest that *SECCE1Rv1G0013110* is a valuable candidate gene conferring resistance to LR and the marker 3596652|F|0–30:A > G-30:A > G is a valuable tool for marker-assisted selection. Apart from this gene, none of the other aforementioned genes were identified as differentially expressed genes in our parallel transcriptome analyses. Nonetheless, many other genes encoding all of the above-mentioned antifungal proteins have been detected [41]. Other genes in this pool, for example, genes encoding an invertase/pectin methylesterase inhibitor family protein, GDSL esterase/lipase, and CHUP1 protein ([42–44], respectively), are associated with fungal pathogens but their role in the response to LR remains unclear. The gene *SECCE1Rv1G0012070*, encoding a dehydrogenase (including glucose and ribitol dehydrogenase) that is categorized among stress-defensive proteins, has been identified *inter alia* in wheat [45]. As many as five of the above-mentioned genes – those coding for an endoglucanase, NBS-LRR disease resistance protein-like protein, pathogenesis-related thaumatin family protein, phenylalanine ammonia-lyase, and MYB-related transcription factor – are located in the region between 96.7 and 137.6 Mb flanked by markers co-segregating with the *Pr3* gene.

However, some genes in this pool (e.g., genes coding for a carboxyl-terminal peptidase, peptide chain release factor 1 or RNA polymerase II subunit B1 CTD phosphatase RPAP2, folylpolyglutamate synthase, and DNA repair helicase), although involved in diverse biological processes, have not previously been assigned a clear function in defense against stresses, including LR. In several genes in this pool that have as yet unconfirmed roles in the immune response to LR, namely, those encoding an invertase/pectin methylesterase inhibitor family protein, CHUP1 protein, RNA polymerase II subunit B1 CTD phosphatase RPAP2, and peptide chain release factor 1, s_DArTseq markers characterized by an extremely high *p*-value are located. Therefore, these genes are potential candidates conferring resistance to LR in rye, although experimental verification is required.

Apart from three markers located in introns of genes encoding a protein phosphatase 2C-like protein, MYB-related transcription factor, and folylpolyglutamate synthase, the remaining markers were located in exons, which in the case of SNPs might cause the synthesis of a distinct protein variant or an inactive protein.

### Predicted translational effects of gene-located SNPs

To predict the potential impact of polymorphisms on the structure (and properties) of proteins, we performed an Ensembl Variant Effect Predictor [31] analysis, which showed that most polymorphisms within exons have low impact on protein structure and properties, and thus cause harmless or insignificant changes. A portion of the SNPs, however, were assessed as causing moderate effects that may result in non-disruptive variant formation. Such variants might change the protein effectiveness. The only polymorphism that was predicted to have a strong translational effect, through loss of the stop codon, was detected in the marker 3594357|F|0–29:A > C-29:A > C located in the gene *SECCE1Rv1G0014450.1*, which codes for a pathogenesis-related thaumatin family protein. The polymorphism A/C results in the change of the TAG stop codon to the GAG codon for glutamic acid and, therefore, elongation instead of termination of the polypeptide chain. This, in turn, must result in loss of protein function. Given that the DArTseq marker genotype in resistant lines is CC, the loss-of-function of an important protein can be assumed from the perspective of the immune response, resulting in increased resistance to the disease. This is a completely unexpected and never previously reported effect. However, although with strong support, verification experiments are required to confirm this hypothesis.

Among markers from the “common 21 pool”, all SNPs in non-coding regions were predicted to have modifier effects without affecting the splicing process.

Excluding the “common 21 pool”, we subjected the remaining s_SNP-DArT markers located in Lo7 genes to an Ensembl Variant Effect Predictor analysis. Similar to the “common 21 pool”, most SNPs had a low translational impact and only one SNP was evaluated to have a high translational impact, namely, that in the *SECCE1Rv1G0016320.1* gene encoding a haloacid dehalogenase-like hydrolase superfamily protein. Similar to the *SECCE1Rv1G0014450.1* gene encoding a pathogenesis-related thaumatin family protein, the effect of stop-codon loss was determined by the recessive allele present in LR-resistant lines. The role of this enzyme in the immune response of plants to fungal pathogens is not yet known. However, such proteins are known to be involved in plant growth and abiotic stress response [46].

Unlike SNPs in non-coding regions in the previous pool, we observed three categories of influence in non-coding sequences, namely, intron variants, downstream gene variants, and upstream gene variants. Thus, there is a broader potential spectrum of possibilities for regulating the expression of genes within which s_SNP-DArT markers are located.

In this study, a GWAS approach, using DArT marker genotyping and detached leaf assay phenotyping of the mapping population constructed by crossing rye lines resistant and susceptible to LR, allowed the detection of two QTLs for the immune response to LR on chromosome 1R. Each QTL was composed of markers strongly associated with the trait of interest; more than half of the markers were located in Lo7 genes, including genes with known functions (among them, *SECCE1Rv1G0013110.1* and *SECCE1Rv1G0014220.1* encode an endoglucanase and NBS-LRR disease resistance protein-like protein, respectively) and genes of currently unknown function (e.g., *SECCE1Rv1G0013840.1* and *SECCE1Rv1G0013960.1* encode an invertase/pectin methylesterase inhibitor family protein and CHUP1 protein, respectively). Most of the predicted translational effects of markers located in gene exons were of low or moderate impact, and those located in introns had modifier effects. Only two SNPs, located in genes that encode a pathogenesis-related thaumatin family protein and a haloacid dehalogenase-like hydrolase superfamily protein, caused loss of the stop codon and were categorized as having a high translational impact, resulting in translation of non-functional proteins. Given the present results and those of other authors, we propose that the gene *SECCE1Rv1G0014220.1*, which encodes a NBS-LRR protein, is the most likely candidate for the *Pr3* gene. In conclusion, by using an approach combining the principles of GWAS and linkage mapping, the present results expand on existing knowledge of the genetic basis of the immune response to LR in rye.

### Supplementary Information

**Supplementary Material 1.**

**Supplementary Material 2.**

**Supplementary Material 3.**

**Supplementary Material 4.**

**Supplementary Material 5.**

**Supplementary Material 6.**

**Supplementary Material 7.**

**Supplementary Material 8.**

**Supplementary Material 9.**

**Supplementary Material 10.**

**Supplementary Material 11.**

**Supplementary Material 12.**

**Supplementary Material 13.**

**Supplementary Material 14.**

**Supplementary Material 15.**

**Supplementary Material 16.**

**Supplementary Material 17.**

**Supplementary Material 18.**

**Supplementary Material 19.**

**Supplementary Material 20.**

**Supplementary Material 21.**

**Supplementary Material 22.**

## Data Availability

The raw RNA-seq (fastq) data are deposited in the NCBI database (BioProject: PRJNA888031). The remainder of the data used in this work is included in the supplementary files.
