# Correction: Identification of quantitative trait loci associated with leaf rust resistance in rye by precision mapping

**DOI:** 10.1186/s12870-024-05072-x

**Published:** 2024-05-11

**Authors:** Mateusz Matuszkiewicz, Agnieszka Grądzielewska, Magdalena Święcicka, Alperen Ozturk, Monika Mokrzycka, Dolapo Igbari Aramide, Jie Song, Andrzej Kilian, Monika Rakoczy-Trojanowska

**Affiliations:** 1 Department of Plant Genetics, Breeding and Biotechnology, Institute of Biology, Warsaw, University of Life Sciences, Warsaw, Poland; 2 Educo BSH Ltd, Lublin, Poland; 3 Graduate School of Science and Technology, Niigata University, Niigata, Japan; 4 Department of Biometry and Bioinformatics, Institute of Plant Genetics Polish Academy of Sciences, Poznań, Poland; 5 Department of Botany, Faculty of Science, University of Lagos, Akoka, Lagos, Yaba Nigeria; 6 Diversity Arrays Technology, University of Canberra, Monana Street, Bruce, ACT 2617 Australia

**Correction** **:** **BMC Plant Biol 24, 291 (2024)**

**https://doi.org/10.1186/s12870-024-04960-6**

Following publication of the original article [1], an error has been introduced in the online publication of the author names. It was found out that the given names and the family names were switched.

The incorrect author names were tagged as follows:

**Table:** 

**Given name**	**Family name**
Matuszkiewicz	Mateusz
Grądzielewska	Agnieszka
Święcicka	Magdalena
Ozturk	Alperen
Mokrzycka	Monika
Igbari Aramide	Dolapo
Song	Jie
Kilian	Andrzej
Rakoczy-Trojanowska	Monika

The correct author name should have been tagged as follows:

**Table:** 

**Given name**	**Family name**
Mateusz	Matuszkiewicz
Agnieszka	Grądzielewska
Magdalena	Święcicka
Alperen	Ozturk
Monika	Mokrzycka
Dolapo	Igbari Aramide
Jie	Song
Andrzej	Kilian
Monika	Rakoczy-Trojanowska

The author group has been updated above and the original article [1] has been corrected.
