# Supplementary material for: Identification of quantitative trait loci associated with leaf rust resistance in rye by precision mapping
Source: BMC Plant Biol. 2024 Apr 17;24:291. doi: 10.1186/s12870-024-04960-6 (PMC11022434; doi:10.1186/s12870-024-04960-6)
Supplement: Supplementary file 18 — Supplementary Material 18. [file 12870_2024_4960_MOESM18_ESM.docx]

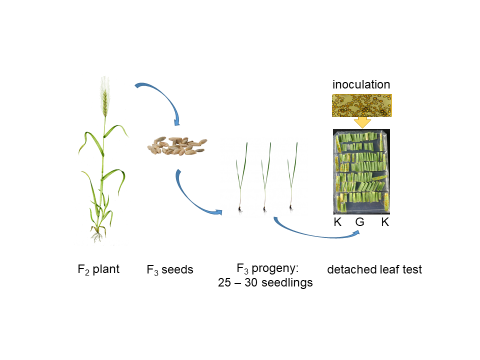


Figure S1. **Experiment design. K – leaf fragments of Konto; G –leaf fragments of F_3_ seedlings derived from G38A mapping population.**
