# Supplementary material for: Identification of quantitative trait loci associated with leaf rust resistance in rye by precision mapping
Source: BMC Plant Biol. 2024 Apr 17;24:291. doi: 10.1186/s12870-024-04960-6 (PMC11022434; doi:10.1186/s12870-024-04960-6)
Supplement: Supplementary file 19 — Supplementary Material 19. [file 12870_2024_4960_MOESM19_ESM.docx]

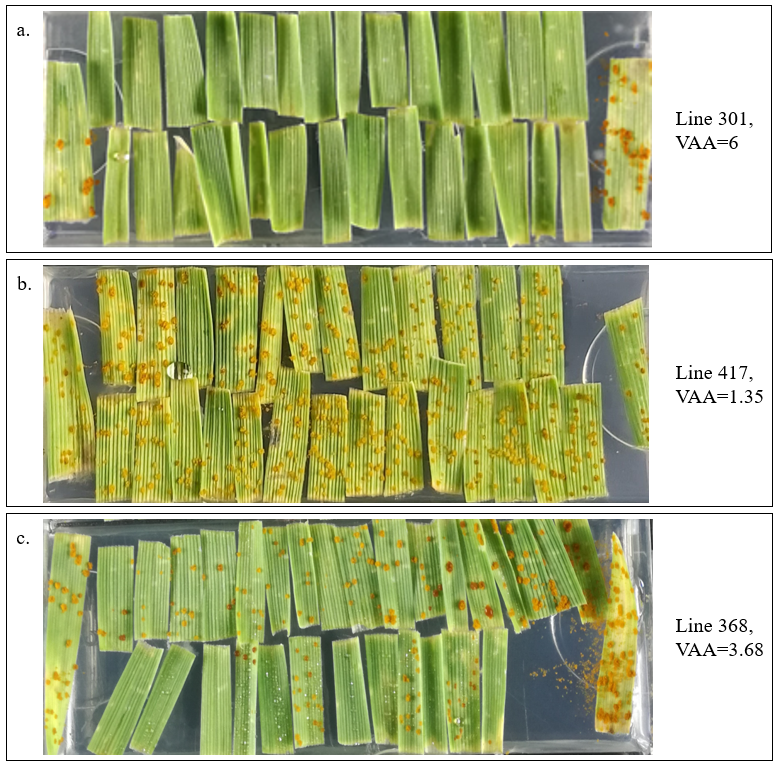


Figure S2. **Detached leaf test of G38A mapping population inoculated with *Prs* isolate.** An examples of phenotypic classes in the mapping population: a. LR-resistant line (VAA ≥ 5), b. LR-susceptible line (VAA ≤ 2.65), c. LR- moderately resistant to moderately susceptible line (4.99≤VAA≥ 2.66).
