# Supplementary material for: Identification of quantitative trait loci associated with leaf rust resistance in rye by precision mapping
Source: BMC Plant Biol. 2024 Apr 17;24:291. doi: 10.1186/s12870-024-04960-6 (PMC11022434; doi:10.1186/s12870-024-04960-6)
Supplement: Supplementary file 20 — Supplementary Material 20. [file 12870_2024_4960_MOESM20_ESM.docx]

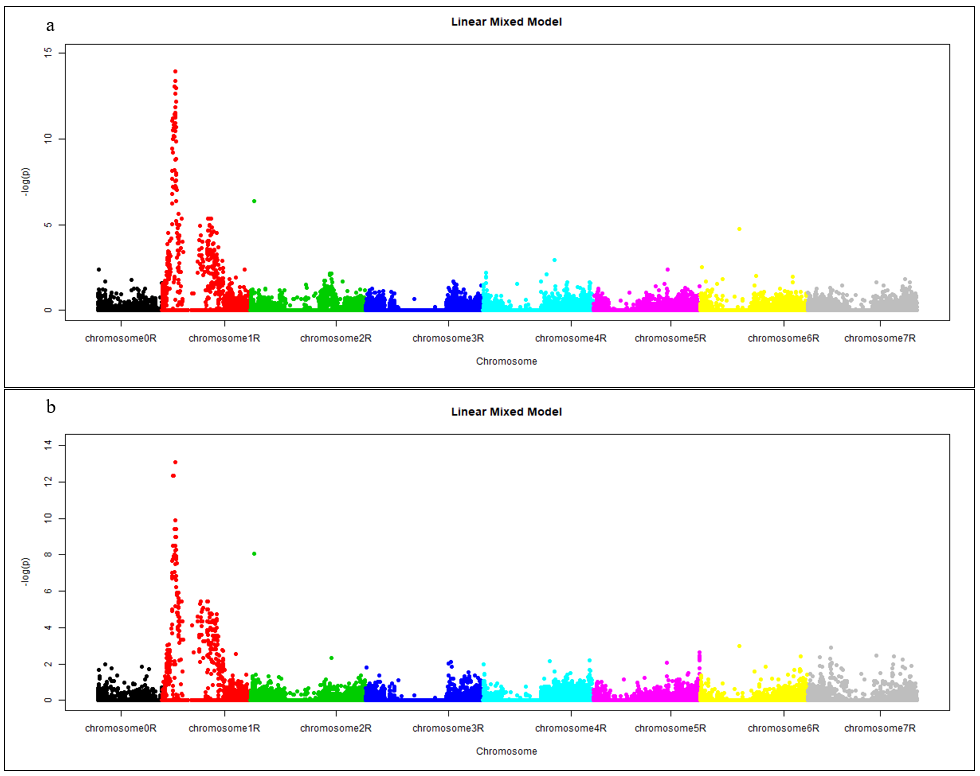


Figure S3. **Manhattan plot visualizing SNP-DArT markers associated with the immune response to leaf rust isolate N, 10 (a) and 17 (b) dpi.**
