# Supplementary material for: Identification of quantitative trait loci associated with leaf rust resistance in rye by precision mapping
Source: BMC Plant Biol. 2024 Apr 17;24:291. doi: 10.1186/s12870-024-04960-6 (PMC11022434; doi:10.1186/s12870-024-04960-6)
Supplement: Supplementary file 21 — Supplementary Material 21. [file 12870_2024_4960_MOESM21_ESM.docx]

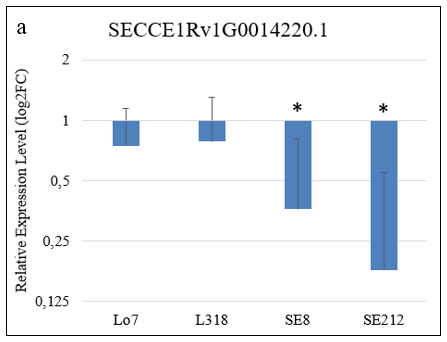


Figure S5a. **Relative expression level (log_2_FC) of the *SECCE1Rv1G0014220.1* gene coding for NBS-LRR disease resistance protein-like protein in rye lines Lo7, L318, SE8 and SE212** (the first two susceptible and the next two - LR resistant) after infection with 1.1/6 *Prs* isolate. The asterisk (*) indicate statistically significant difference with p < 0.05 . The sequences of primers used in RT-qPCR are listed in the Table S16. RNA isolation, cDNA synthesis and RT-qPCR reaction was performed according to Święcicka et al. Changes in benzoxazinoid contents and the expression of the associated genes in rye (*Secale cereale* L.) due to brown rust and the inoculation procedure. PLoS One. 2020;15(5):e0233807.


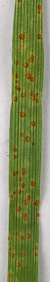

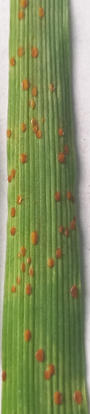

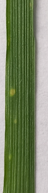

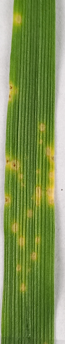


Lo7

L318

SE8

SE212

4

4

2

1

Figure S5b. **Macroscopic examination of LR infection symptoms on rye leaves of lines Lo7, L318, SE8 and SE212 infected with 1.1/6 *Prs* isolate, 10 dpi.** The infection types determined using the following 0 – 5 scale (Murphy, 1935) are provided in parentheses: 0 = immune (no visible reaction), 1 = very resistant (chlorotic and necrotic flecking), 2 = resistant (minute uredinia, surrounded by chlorosis or necrosis), 3 = resistant to moderately resistant (small to medium-size uredinia, surrounded by chlorosis or necrosis), 4 = moderately resistant to moderately susceptible (medium to large uredinia, surrounded by chlorosis), and 5 = susceptible (large uredinia without chlorosis).
